# Xenogenic Corneal Lenticules (XENIA) – Biomechanical Characterization and Enzymatic Resistance Analysis

**DOI:** 10.1167/tvst.14.12.34

**Published:** 2025-12-31

**Authors:** Stephan Reiter, Joana Witt, Johannes Menzel-Severing, Gerd Geerling, Theo G. Seiler

**Affiliations:** 1 Department of Ophthalmology, Medical Faculty and University Hospital Düsseldorf, Heinrich-Heine-University Düsseldorf, Germany; 2 Institut für Refraktive und Ophthalmo-Chirurgie (IROC), Zürich, Switzerland; 3 Universitätsklinik für Augenheilkunde, Inselspital Bern, Bern, Switzerland

**Keywords:** cornea, xenogenic, XENIA, implant, lenticule, enzymatic digestion, stress-strain measurements

## Abstract

**Purpose:**

Decellularized and cross-linked porcine stromal tissue offers a new option for corneal tissue augmentation. The aim of this study is to characterize the biomechanical properties and enzymatic resistance of processed xenogenic porcine corneal lenticules (XENIA) and compare them to human/porcine specimens.

**Methods:**

Groups of XENIA, human, and porcine corneal lenticules were formed. The dimensions of all lenticules were 7.7 mm in diameter and 80 µm in thickness. Conformité Européenne (CE)-approved XENIA-lenticules were provided by the manufacturer and human/porcine lenticules were generated using a femtosecond laser from the anterior stroma of cadaver eyes. All specimens were analyzed by uniaxial stress-strain measurements (*n* = 6 per group) and the resulting elastic moduli were compared. To evaluate the enzymatic resistance, all lenticules (*n* = 5 per group) were treated with collagenase solution (c = 0.1 U/mL) over a 20-day period and the lenticule size was analyzed by photo-documentation.

**Results:**

XENIA-lenticules showed the highest elastic modulus, significantly higher than human and porcine lenticules. At 11% strain, the maximum difference occurred between XENIA and porcine lenticules, with XENIA-lenticules being 23.5-fold stiffer. In the human lenticule group, donor age correlated strongly with the elastic moduli of this group (*r*_s_ = 0.941, *P* = 0.005). XENIA-lenticules showed the greatest resistance against enzymatic digestion. On average, human samples were completely digested after 11 ± 4 hours, porcine samples after 91 ± 60 hours, whereas XENIA-lenticules only showed a digested area of 19.2% ± 13.7% after 20 days.

**Conclusions:**

XENIA-lenticules are substantially stiffer (3.9–9.6-fold stiffer than human tissue and 9.0–23.5-fold stiffer than porcine tissue across 5% to 17% strain) and resistant to enzymatic digestion compared to human/porcine probes.

**Translational Relevance:**

XENIA-lenticules may help to overcome the lack of human corneal donor tissue and may offer new alternatives for corneal augmentation therapies, such as for corneal melting or keratoconus.

## Introduction

The shortage of human donor tissue is considered a major problem in modern health care.^1^ In the past, approaches with xenogenic material have been assessed to overcome this shortage, such as with cardiac or aortic valve replacements^2^ or, recently, even with entire heart transplants.^3^ The lack of donor tissue is also problematic in ophthalmology and one reason why therapies for corneal pathologies with progressive thinning do not include tissue augmentation as a standard clinical procedure yet. In particular, corneal stromal melting or advanced keratoconus are difficult and challenging conditions with only limited therapeutic options. Xenogenic corneal tissue may offer a solution for this shortage and in the past decade new xenogenic approaches have been undertaken with the use of acellular porcine corneal stroma.^4^^–^^6^ Recently, Gebauer Medizinprodukte GmbH (Neuhausen, Germany) received CE- and MDR-approval for its xenogenic corneal implant (XENIA). It is obtained from the anterior section of a porcine cornea and then further processed. In a first step the corneas are decellularized to remove antigenic epitopes and DNA, followed by a washing process to remove Gal-complexes. Subsequently, the corneas are compressed and finally intensively cross-linked by actinic radiation.^7^

A recent study conducted by Wilson et al.^7^ focused on biomechanical properties of XENIA-lenticules using displacement speckle pattern interferometry (DSPI) to investigate the effect of the washing and stiffening process on the mechanical strength of XENIA-lenticules in comparison to non-processed porcine lenticules. Their findings indicate an increase in stiffness by 127%. However, a biomechanical comparison to human tissue, as well as the resistance to enzymatic digestion has not been investigated yet for XENIA-lenticules. Understanding the enzymatic resistance of such additive lenticules or ring segments is particularly crucial in conditions like corneal melting and progressive thinning, such as in keratoconus, in the context of which it is well known that enzyme levels of matrix metalloproteinases are elevated^8^^,^^9^ and contribute to disease progression.^10^ Because the XENIA samples are treated by an intense cross-linking procedure, increased resistance can be expected based on previous corneal cross-linking experiments.^11^

Therefore this study aims to investigate the biomechanical properties and enzymatic resistance of XENIA-lenticules compared to non-processed human and porcine controls. The findings will provide valuable insights into the potential of xenogenic corneal lenticules for tissue augmentation, proposing new therapeutic approaches for conditions like corneal melting and advanced keratectasia.

## Material and Methods

### Corneal Tissue

**Figure 1.**
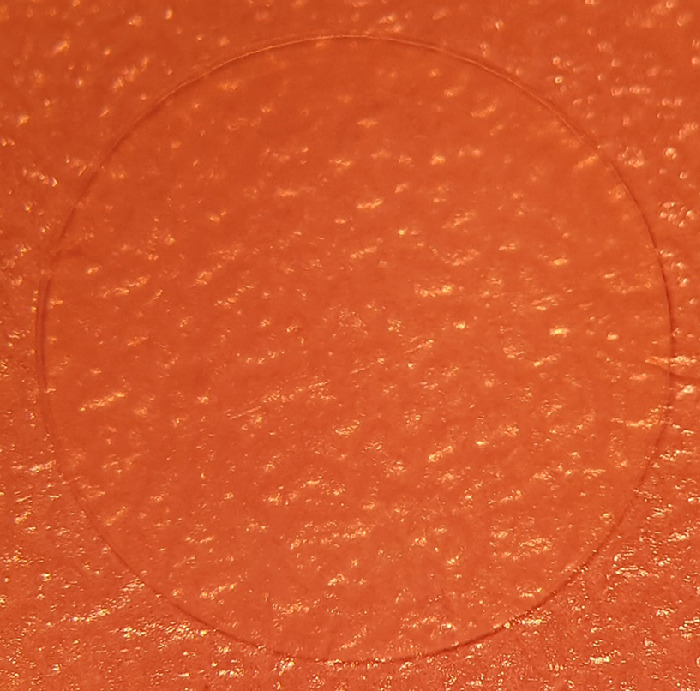
XENIA-lenticule before preparation.

Eleven processed and artificially stiffened porcine xenogenic lenticules (XENIA) were provided by the manufacturer in a storage medium composed of glycerol, water, and ethanol. The CE-marked and MDR-approved XENIA-lenticules were planar and circular-shaped discs of 7.7 mm in diameter and 80 µm in thickness (Fig. 1). They were harvested from the anterior part of the cornea including Bowman's layer. The pretreatment of the XENIA-lenticules included a decellularization process to remove antigenic epitopes and DNA to minimize the risk of immunological reactions, washing, compression, and an intensive cross-linking procedure with actinic radiation to increase their stiffness.^7^^,^^12^ At the time of the experiments, the stiffness was standardized for all CE-marked lenticules and could not be varied. For the second group, eleven human corneas (donor age: 78 ± 8 years, eight female and three male individuals) were allocated by the LIONS Eye Bank NRW (University Hospital Düsseldorf, Heinrich-Heine-University, Germany) after a storage time between seven to 28 days. Before the experiments, Descemet's membrane, including the endothelium, was peeled off and used for DMEK surgery without damaging the corneal stroma. Between the peeling and further processing, the corneas were kept for a maximum of 12 hours in organ culture medium containing 6% dextran to prevent corneal swelling according to the current organ culture storage solution guideline.^13^ The use of the donor tissue was approved by the local institutional review board and the relatives had agreed to the scientific use of the tissue. For the third group, eleven porcine corneas were obtained from a local abattoir. The eyes were transported in phosphate-buffered saline solution (Sigma-Aldrich, St. Louis, MO, USA) and processed within 12 hours postmortem. Because all stress-strain-measurements were performed in an unphysiological biomechanical range, enzymatic resistance analysis was conducted on different samples to avoid bias from potential tissue destruction. All experiments were performed according to the Declaration of Helsinki.

### Lenticule Creation From Non-Processed Human and Porcine Corneas

**Figure 2.**
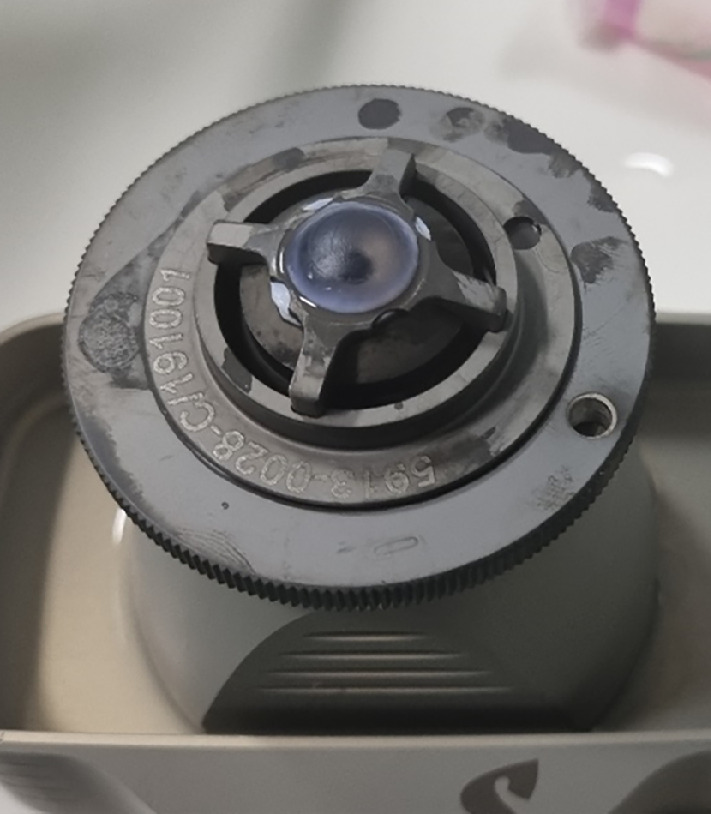
Human cornea mounted in an artificial anterior chamber, after the generation of the lenticule.

The lenticule creation was performed with an OCT-controlled femtosecond laser (LDV Z8; Ziemer Ophthalmic Systems AG, Port, Switzerland). After epithelial removal, human corneas were mounted in an artificial anterior chamber to generate the lenticule (Fig. 2), whereas for porcine lenticule creation, entire globes were used. All lenticules were dissected from the anterior corneal stroma using an applanating mode including Bowman's layer with a diameter of 7.7 mm and 80 µm in thickness in a planar geometry to ensure equal biomechanical testing conditions. Although in recent years more evidence was found for the existence of Bowman's layer in porcine corneas, the biomechanical impact is still unclear, as well in human corneas.^14^^–^^16^ Fiber orientation was not taken into account as the provided XENIA-lenticules are not orientated. After the dissection, lenticules were placed in 2 mL test tubes containing organ culture medium with 6% dextran to maintain corneal hydration.

### Preparation of Processed Porcine Lenticules (XENIA)

Before the experiments, human and porcine lenticules were rinsed with balanced salt solution (BSS) (Deltamedica, Reutlingen, Germany). The XENIA-lenticules were treated according to the manufacturer's guidelines by shaking at 500 rpm in BSS for three intervals of 15 minutes each. For better visualization, a drop of 0.4 % trypan-blue solution (Sigma-Aldrich, St. Louis, MO, USA) was applied to the surface of all lenticules.

### Uniaxial Stress-Strain Measurement

**Figure 3.**
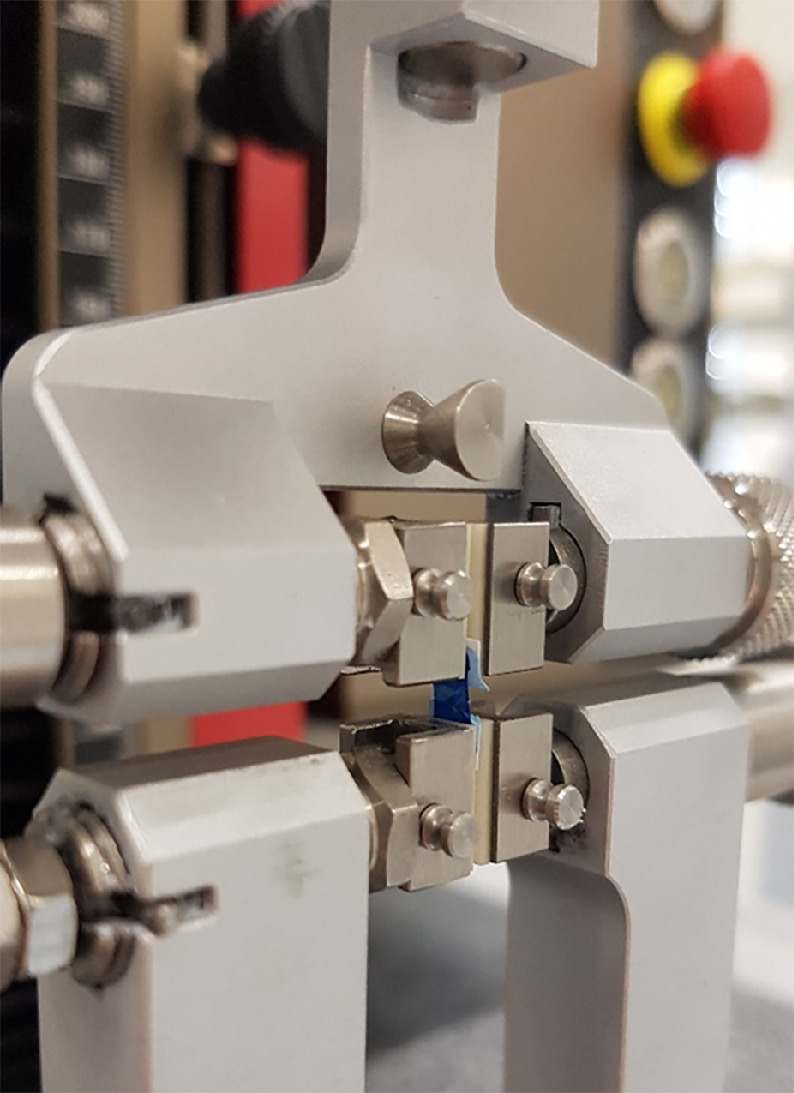
Corneal lenticule strip between the clamps of the material tester.

For stress-strain measurements of the lenticules (*n* = 6 per group), central simple strips of 5 mm width according to similar experiments^15^^,^^17^^,^^18^ were excised using two fixed, parallel-aligned razor blades. The measurements were performed using the material testing device ZwickiLine (Zwick Roell, Ulm, Germany) with the load cell XForceP (nominal force 10 N, resolution 0.04 N). Strips were clamped between two jaw pairs consisting of oxide ceramics with a clamp distance of 2.5 mm (Fig. 3) and slippage was checked. To ensure equal starting conditions, a prestress of 10 kPa was applied to all mounted specimens. The stress-strain cycle was performed at a velocity of 2 mm/min until a strain of 19%, similar to previous experiments.^17^ The data was recorded with testXpert III software (Zwick Roell) and exported to a Microsoft Excel file (Microsoft Corporation, Redmond, WA, USA) including the absolute strain, the x-corrected strain, the force, and the distance. The elastic modulus (Young's modulus, $E=\frac{\sigma }{ɛ}$) was calculated for each sample at 5%, 8%, 11%, 14%, and 17% with a strain window of ±0.04%.

### Digestion Resistance to Collagenase

A total of 15 lenticules (*n* = 5 per group) were placed into collagenase A solution (enzyme activity 0.223 U/mg, c = 0.1 U/mL in phosphate-buffered saline solution from *Clostridium histolyticum* (LOT 35830424; Roche, Basel, Switzerland), and the digestion process was observed over 20 days, similar to previous studies.^11^ The resistance tests were performed in 24-well plates with 500 µL of the prepared collagenase solution. To document the digestion process, photographs of all lenticules were taken with a digital camera on a white background. The well plate diameter of 15.22 mm served as reference scale and was always completely displayed in the photos. The collagenase solution was replaced every 24 hours.

**Figure 4.**
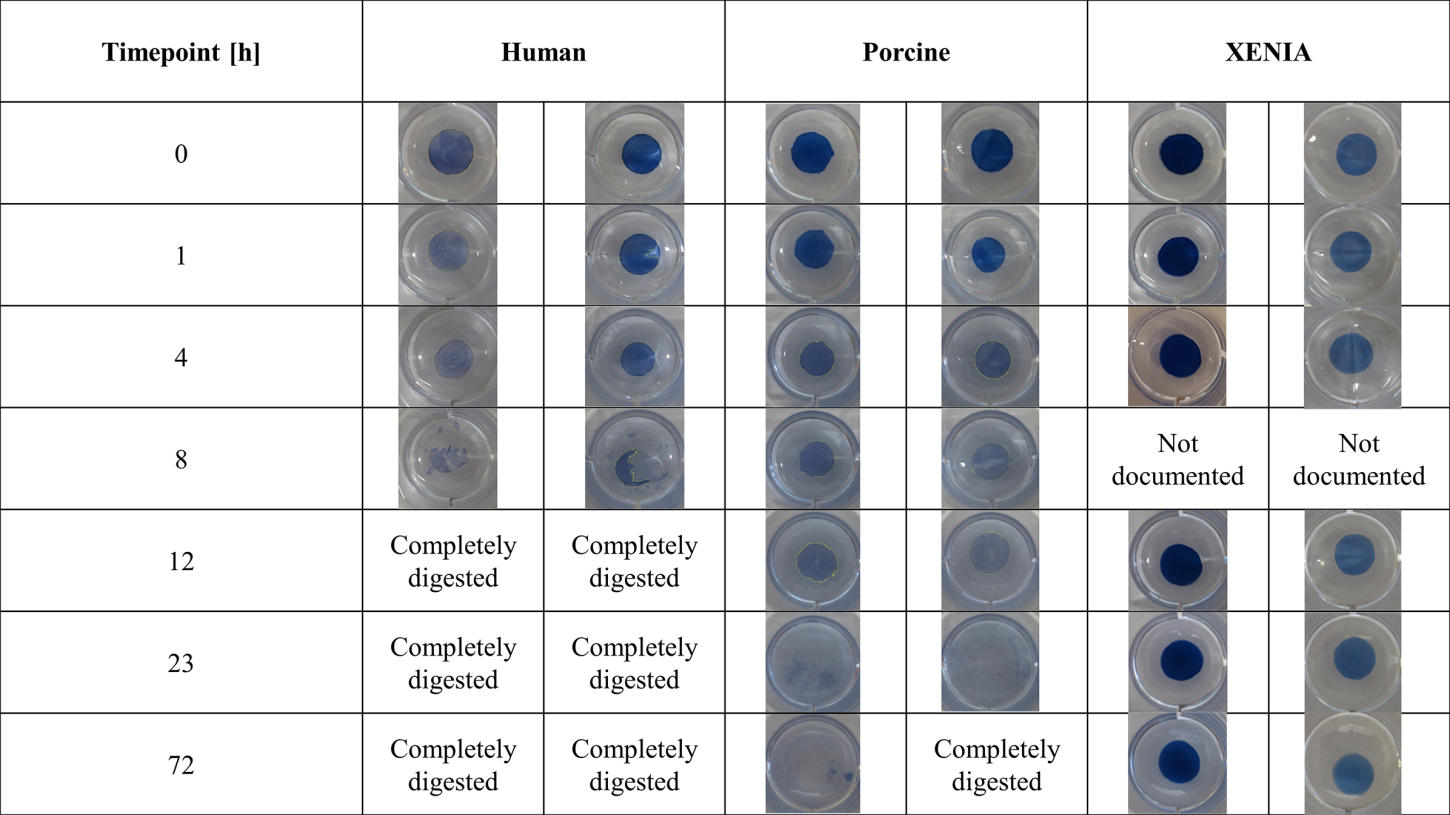
Photo-documentation of the digestion process of human, porcine, and XENIA-lenticules at representative timepoints. Exemplary two lenticules of each group are shown here.

After the first photo-documentation, the well plate, including the samples, was placed in an incubator at 37°C temperature. At each time point, the well plate was removed from the incubator and the photo-documentation was repeated (Fig. 4). Overall, 21 time points were documented (Table 1) over the course of 20 days, and the tests were stopped independently of the remaining lenticule size. The examined parameter was the lenticule area. The first photographed area of each lenticule was defined as 1 (100% remaining area) and the following areas were defined as the percentage of the remaining area. The photos were analyzed with ImageJ software (National Institutes of Health, Bethesda, MD, USA). After the diameter of the wells displayed in the photos was set manually as a reference scale, the circumference of each lenticule was determined based on the visible rim of each sample. The resulting area size of each lenticule was calculated automatically from the given values by ImageJ.

**Table 1.** Digestion Over Time of Human, Porcine, and XENIA-Lenticules

Timepoint	Human	Porcine	XENIA
1 (0 h)	100.0% ± 0.0%	100.0% ± 0.0%	100.0 ± 0.0%
2 (1 h)	94.4% ± 6.7%	92.0% ± 8.7%	101.0% ± 3.0%
3 (2 h)	76.0% ± 16.5%	92.2% ± 3.7%	98.0% ± 5.0%
4 (3 h)	63.2% ± 25.9%	86.8% ± 6.6%	93.4 ± 4.2%
5 (4 h)	59.6% ± 23.7%	85.5% ± 5.3%	94.0%± 3.9%
6 (5 h)	54.6% ± 30.8%	83.2% ± 9.3%	Not measured
7 (6 h)	53.6% ± 30.0%	82.5% ± 7.1%	Not measured
8 (7 h)	46.0% ± 26.2%	81.2% ± 10.1%	Not measured
9 (8 h)	41.0% ± 23.2%	85.0% ± 7.7%	Not measured
10 (9 h)	46.2% ± 27.6%	81.5% ± 10.6%	Not measured
11 (10 h)	38.8% ± 22.6%	85.0% ± 12.5%	Not measured
12 (11 h)	7.0% ± 15.7%	90.7% ± 10.7%	Not measured
13 (12 h)	6.2% ± 13.9%	92.5% ± 13.8%	Not measured
14 (17 h)	0.0%	87.3% ± 17.5%	92.8% ± 7.8%
15 (20 h)	0.0%	81.7% ± 47.4%	90.6% ± 6.2%
16 (23 h)	0.0%	64.5% ± 51.2%	92.2% ± 5.4%
17 (3 d)	0.0%	16.2% ± 19.2%	91.2% ± 4.9%
18 (6 d)	0.0%	0.0%	87.8% ± 6.0%
19 (10 d)	0.0%	0.0%	84.2% ± 9.3%
20 (13 d)	0.0%	0.0%	83.8% ± 12.1%
21 (20 d)	0.0%	0.0%	80.8% ± 13.7%

The remaining area is displayed as mean percentage of the initial standardized area (mm²) at timepoint 1 (0 hours). The digestion process was stopped after 20 days, independently of remaining lenticule samples.

### Statistical Evaluation

All values are expressed as mean value ± standard deviation including 95% confidence interval (CI) wherever biologically appropriate. For stress-strain measurements, elastic moduli were compared using two-way analysis of variance. Correlation between the donor age of the human samples and the elastic moduli for each strain was calculated with Spearman's rank correlation. For enzymatic digestion, the percentages of digested and non-digested lenticules were compared using χ^2^ test. Furthermore, the percentage remaining areas per time point of each lenticule group were compared with a two-way analysis of variance. For multiple comparisons of elastic moduli, Bonferroni correction was used (Bonferroni-adjusted α = 0.003). Statistical analysis was performed with the SPSS software Version 28.0.0.0 (IBM, Armonk, NY, USA). Significance was accepted for *P* values < 0.05.

## Results

**Figure 5.**
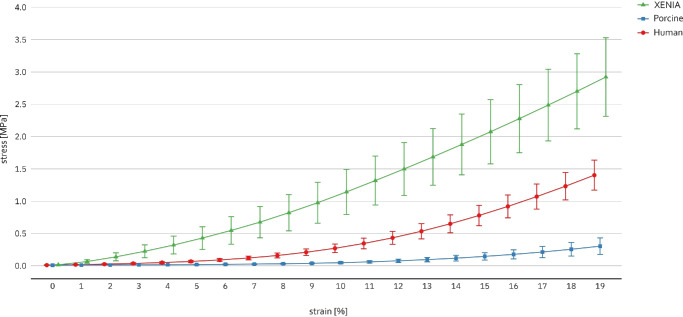
Stress-strain-curves of human, porcine and XENIA-lenticules up to 19% strain. Young's modulus was calculated as tangent modulus E = σ/ε within a strain window of ± 0.04%.

The stress-strain extensiometry measurements of the corneal lenticules showed the typical nonlinear elastic behavior of biological tissues, as depicted in Figure 5. The elastic moduli of the three groups are listed in Table 2. XENIA processed porcine lenticules were significantly stiffer than human and porcine lenticules at all analyzed strains (*P* < 0.001). At 5% strain, the elastic modulus of XENIA-lenticules were nine times higher (95% CI, 4.4–17.9] than that of unprocessed porcine lenticules and seven times higher (95% CI, 2.1– 22.2) compared to human lenticules. At 11% strain, this factor increased to 23.5-fold (95% CI, 14.7–37.6) compared to the unprocessed porcine group. Surprisingly, human and porcine lenticules showed no significant difference at 5% and 8% strain (*P* = 0.595 and *P* = 0.1) but at 11%, 14%, and 17%, when the elastic modulus of the human corneas was significantly higher (Table 2). For the six human specimens, there was a significant positive correlation between the donor age of the human samples and the elastic moduli (*r*_s_ = 0.941, *P* = 0.005) at all examined strains.

**Table 2.** Elastic Moduli (E, MPa) of Human, Porcine, and XENIA-Lenticules at 5%, 8%, 11%, 14%, and 17% Strain

Type of Lenticule	E at 5%	E at 8%	E at 11%	E at 14%	E at 17%
Human	0.36 ± 0.38 [0.06; 0.66]	0.52 ± 0.34 [0.25; 0.79]	0.90 ± 0.36 [0.61; 1.19]	1.62 ± 0.45 [1.26; 1.98]	2.76 ± 0.63 [2.26; 3.26]
Porcine	0.28 ± 0.04 [0.25; 0.31]	0.26 ± 0.06 [0.21; 0.31]	0.30 ± 0.08 [0.24; 0.36]	0.46 ± 0.11 [0.37; 0.55]	0.71 ± 0.18 [0.57; 0.85]
XENIA	2.48 ± 1.62 [1.18; 3.78]	4.98 ± 2.61 [2.89; 7.07]	7.05 ± 2.98 [4.67; 9.43]	8.97 ± 3.13 [6.47; 11.47]	10.64 ± 3.13 [8.14; 13.14]

The elastic modulus was calculated as E = σ/ε within a strain window of ± 0.04%. 95% confidence interval in brackets.

**Figure 6.**
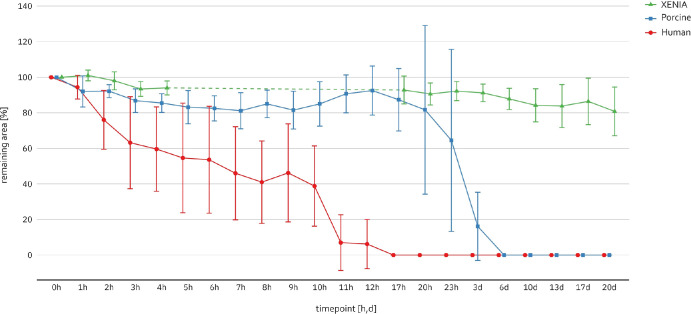
Enzymatic digestion of human, porcine and XENIA-lenticules over time up to 20 days. The remaining area of each timepoint is defined as percentage of the initial area at timepoint 0 h (1 = 100%). Enzymatic solution was replenished each 24 hours to maintain stable digestion activity. The *dashed line* indicates timepoints/intervals which were not measured. After 20 days the digestion process was stopped independently of not fully degraded samples.

To evaluate digestion of the lenticules over time, the percentages of digested areas of lenticules were compared at each time point (Table 1). Figure 6 shows that all groups experienced an area reduction over time, however, the digestion process was substantially faster in the human and non-processed porcine group. In the XENIA group, the average area reduction after 20 days was only 19.2% (95% CI, 7.2–31.2), whereas similar values were observed already after two hours in the human group (24.0% reduction [95% CI, −44.5 to −3.5]) and after seven hours in the porcine group (18.8% reduction [95% CI, −31.4 to −6.3]). The average time for the complete digestion was 11.00 ± 4.24 (95% CI, 7.28–14.72) hours in the human group and 91.17 ± 60.27 (95% CI, 42.56–139.78) hours in the unprocessed porcine group, showing a significant difference (*P* = 0.012), whereas none of the XENIA samples showed complete digestion within the 20-day observation period (*P* < 0.001). Bonferroni-adjusted comparisons of all three groups showed significant differences between the XENIA and human group as early as after two hours (*P* = 0.011), whereas a significant difference between the XENIA and porcine group was only observed after three days of digestion (*P* < 0.001). Comparing human and porcine samples, a significant difference occurred after seven hours, with 46.0% (95% CI, 23.0; 68.9) remaining area in the human group versus 81.2% (95% CI, 72.4– 90.1) in the porcine group (*P* = 0.047).

## Discussion

The main findings of this study are (1) that XENIA-lenticules are significantly stiffer and (2) have a significantly higher resistance to enzymatic digestion compared to human and non-processed porcine lenticules. To our knowledge, this is the first study assessing the enzymatic resistance of XENIA-lenticules and testing their biomechanical properties using stress-strain extensiometry.

The stress-strain measurements yielded typical nonlinear elastic stress-strain curves for all corneal lenticules comparable to previous studies.^18^^–^^20^ In a recent study, Wilson et al.^7^ investigated the changes in biomechanical properties of XENIA-lenticules in comparison to virgin porcine lenticules using DSPI. An increased stiffness of 2.3-fold was reported for the XENIA-lenticules. We could show that at strains up to 17%, XENIA-lenticules have an increase in stiffness between four- to ninefold compared to human lenticules and nine- to 23-fold compared to untreated porcine lenticules. We interpret the difference in the increase of stiffness, found in this study compared to results by Wilson et al.^7^ as caused by the different acquisition techniques (DSPI vs. extensiometry)—although we acknowledge the direct comparability of these testing methods is somehow limited—and notably the different strain ranges in which the experiments were conducted. It is remarkable, that the specific processing of virgin porcine corneas to XENIA-lenticules provides such a substantial increase in cornea stiffness, which is an order of magnitude higher than what is known from standard UV-riboflavin CXL.^17^ However, this could be kind of expected by the design of the lenticules because the cross-linking procedure is far more intense than regular CXL and the lenticules are additionally compressed. In a side analysis, we found a positive correlation between the donor age of the human corneas and the elastic modulus, also consistent with the published literature showing the effect of age-depended glycation-mediated natural cross-linking.^21^

Enzymatic activity plays a key role in the pathogenesis of corneal melting as well as in keratoconus, leading to corneal thinning and disease progression.^22^^,^^23^ Therefore requirements on corneal stromal implants should comprise a sufficient stability against proteolytic enzymes. Our results show that the resistance of the XENIA-lenticules was significantly higher than in the control groups. We anticipate this as a consequence of the strong cross-linking performed by actinic radiation during the generation of the XENIA-lenticules. To our knowledge, there are no studies investigating enzymatic digestion of XENIA-lenticules or other corneal lenticules, as only full-thickness corneas were evaluated. In 2021, Donovan et al. investigated the enzymatic digestion of full-thickness human corneas, treated with the chemical cross-linker Genipin versus human controls. Their results showed that untreated corneal buttons of 2 mm in diameter were completely digested by 1% collagenase solution within four hours, whereas the Genipin-treated samples needed seven hours^24^ close to the digestion time of human corneas of 11 hours in the present study. Similar results were found by Lammer and coworkers^25^ for human samples. We assume that the difference in digestion time arises from the different dimensions of the tested specimen and the different collagenase solution concentration. Regarding porcine lenticules, Spoerl et al.^11^ examined 8 mm porcine corneal buttons, which were either untreated or cross-linked with UV-riboflavin using different energy intensities and exposed to 0.1% collagenase solution. The untreated controls were completely digested within six days, which is in agreement to the four days found in this study for the thinner lenticules. Their findings also revealed an effect of UV-riboflavin cross-linking on the cornea against enzymatic digestion as all cross-linked control groups needed significantly longer to be fully digested. Although XENIA-lenticules are cross-linked in a different manner, the even higher increased resistance of the lenticules compared to the control groups is impressive with an order of magnitude more and similar to magnitude of the stiffening effect.

Focusing on a potential clinical application of XENIA-lenticules, there are currently only two studies^12^^,^^26^ that investigated the implantation of XENIA-lenticules in moderate to severe keratoconus. El-Massry et al.^12^ reported seven cases after XENIA-lenticule implantation, in which all eyes tolerated the lenticule well without any immunological reaction after one year. They also reported corneal curvature regularization in absence of adverse events. However, visual acuity improvements were limited to a maximum of 20/50 (except one eye that achieved 20/30) and a potential visual acuity limitation because of reduced light scattering or transmission, evaluated by rigid contact lens visual acuity testing, were not performed. Similar results were found by Deshmukh et al.^26^ who implanted XENIA-lenticules in nine young patients that lead to an improvement of UDVA from 1.43 ± 0.3 to 0.78 ± 0.17 logMAR and CDVA from 0.89 ± 0.13 to 0.45 ± 0.03 logMAR after one year. In contrast to El Massry et al.,^12^ they reported two eyes with a stromal melt 3 months postoperatively and the lenticules needed to be explanted. Also a recent case report by Berger et al.^27^ describes a severe ulcerative keratitis eight months after XENIA-lenticule implantation. The authors suspected a reduced diffusion of glucose through the XENIA-lenticule with consecutive malnutrition of the overlying host cornea leading to the central ulceration.^27^ Based on the substantial increased stiffness and resistance to enzymatic digestion found in this study, other potential clinical applications for XENIA-lenticules, although with different dimensions, are corneal ulcers with stromal melting or corneal augmentation with XENIA-rings segments (e.g., in keratoconus or pellucid marginal degeneration) similar to the approach of corneal allogenic intrastromal ring segments.^28^

Other studies^5^^,^^29^ investigated the used of non-cross-linked acellular porcine corneal stroma for therapeutic lamellar keratoplasty with promising results regarding immunological reactions, visual improvement and adverse events. Recently, an artificial cornea stroma construct imitation created from porcine collagen and processed by chemical cross-linking yielded remarkable visual and graft survival results for lamellar stromal augmentation in keratoconus corneas.^30^ In contrast to XENIA-lenticules, the investigated corneal implant showed three- to fivefold weaker biomechanical properties and at least twice less resistance to enzymatic digestion than human specimen.

Based on our results, published literature and the CE-mark and MDR-approval, XENIA-lenticules may offer a future treatment option in corneal melting or progressed keratectasia. One concern that arises from the study is the biomechanical interaction at the interface of the XENIA-lenticule and the recipient cornea due to the high difference in stiffness similar to findings from intracorneal ring segments. In intracorneal ring segments, biomechanical stresses on the stroma or alterations in the diffusion of metabolites to the stroma overlying the PMMA segment has been described as potential sources of complications.^31^ In addition, before clinical routine implantation in humans, the optimal dimensions and shape of the XENIA-tissue have to be further adjusted to the individual clinical situation.

Concerning limitations of our study, we acknowledge that we applied different protocols for the pretesting preparation to the XENIA-lenticules compared to human and porcine samples in accordance with the manufacturer's guidelines, including prolonged shaking in BSS. However, because the difference in biomechanical properties between XENIA and the other two lenticule groups is very distinctive, we don't expect a major influence of the preparation method on the principial results. The width-to-gauge length of 5 mm/2.5 mm is different from established protocols, because we applied a prestress of 10 kPa for all lenticules to ensure comparability of all samples. We did not perform any preconditioning cycles. With reference to the enzymatic digestion analysis rim-based area measurement in fact does not take into account internal digestion, so alternative approaches like drying, followed by weighing of residual tissue need to be considered as alternatives as performed by O'Brart et al.^32^ and Aldahlawi et al.^33^ However, in the mentioned experiments, dry weight measurements were only performed in a small subset of samples after 10 to 13 days which was not feasible for us due to the very limited availability of XENIA samples.

In summary our results indicate that XENIA-lenticules have several advantages compared to porcine and human non-cross-linked control lenticules with respect to biomechanics and resistance against enzymatic digestion under the in vitro conditions tested here. Furthermore, these xenogenic lenticules require no special storage conditions with almost unlimited availability, which offer an approach to overcome the shortage of human graft tissue. However, further experimental and clinical research is needed to evaluate results and investigate potential indications for XENIA-implantations (e.g., deep corneal ulcers and corneal melting).
